# Garlic peel extract as an antioxidant inhibits triple‐negative breast tumor growth and angiogenesis by inhibiting cyclooxygenase‐2 expression

**DOI:** 10.1002/fsn3.4320

**Published:** 2024-07-09

**Authors:** Yushi Dong, Jiyue Zhang, Aijun Xie, Xiqing Yue, Mohan Li, Qian Zhou

**Affiliations:** ^1^ College of Food Science Shenyang Agricultural University Shenyang China; ^2^ Spice and Beverage Research Institute, Chinese Academy of Tropical Agricultural Sciences Wanning China; ^3^ Department of Chemical and Biomolecular Engineering National University of Singapore Singapore Singapore

**Keywords:** 4T1, antioxidant, COX‐2, garlic peel, triple‐negative breast cancer

## Abstract

Garlic peels are frequently disposed of as agro‐waste; their bioactivity and physiological activity for health benefits and disease protection are neglected. This study aims to examine the potential inhibitory effects of garlic peel extract as an antioxidant on 4 T1 triple‐negative breast cancer (TNBC) tumors in mice. The bioactive constituents of garlic peel were identified through HPLC‐MS/MS analysis, while the antioxidant properties of garlic peel extract were assessed using peroxyl radical scavenging capacity (PSC) and cellular antioxidant activity (CAA) assays. Subsequently, the inhibitory effects of garlic peel extract on 4T1 tumor growth were evaluated using a 4T1 model. The results showed that 433 polyphenol compounds were found in garlic peel extract; among them, flavonoids and phenolic acid are the primary polyphenols with natural antioxidant activity, and both high and low concentrations of the extract exhibited tumor‐suppressive effects. Immunohistochemistry was employed to assess the expression levels of COX‐2, CD31, VEGFA, MMP2, and MMP9 in tumor tissues in order to investigate the antioxidant properties of garlic peel extract, specifically its ability to suppress COX‐2 expression. The findings of this study offer a foundation for the advancement of garlic peel‐based functional products and contribute to the identification of potential anti‐cancer agents and therapeutic targets.

## Graphical abstract

This study demonstrated that garlic peel extract as an antioxidant inhibits triple‐negative breast tumor growth and angiogenesis by inhibiting cyclooxygenase‐2 expression.

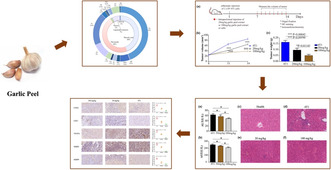

## 1 INTRODUCTION

Breast cancer is the primary cause of cancer‐related mortality among women (DeSantis et al., 2019; Li et al., 2021). According to the National Comprehensive Cancer Network Breast Cancer Clinical Practice Guidelines, triple‐negative breast cancer (TNBC) is characterized by the absence of estrogen and progesterone receptors, as well as low expression of human epidermal growth factor receptor 2 (Bevers et al., 2009; Li et al., 2020). TNBC is identified as the most aggressive subtype, exhibiting advanced histological grade and unfavorable clinical outcomes even with appropriate therapeutic interventions (Li et al., 2022; Reis‐Filho & Tutt, 2008). Research has indicated a strong correlation between oxidative stress and the incidence and progression of breast cancer (Basnet et al., 2019; Diehn et al., 2009; Li, Li, et al., 2021). Oxidative stress is a process of activation of cancer‐promoting signaling due to the loss of intracellular oxidative homeostasis caused by the explosion of reactive oxygen species (ROS) (Acharya et al., 2010; Shen et al., 2024). In the process of oxidative stress, cyclooxygenase 2 (COX‐2) is a key rate‐limiting enzyme involved in arachidonic acid metabolism and regulated by ROS (Singh et al., 2007). Studies have shown that the high expression of COX‐2 is closely related to the occurrence of breast cancer because tumor cells can maintain high levels of ROS by overexpressing COX‐2 (Hou et al., 2011), leading to the promotion of tumor progression by increasing MMP2 (Sivula et al., 2005) and MMP9 expression (Liou & Storz, 2010; Mohammad et al., 2012) and excessive angiogenesis by increasing VEGFA protein synthesis and secretion (Davies et al., 2003; von Rahden et al., 2005).

Plants produce a large amount of oxidants during photosynthesis (Blando et al., 2019; Foyer & Noctor, 2003; Xie, Dong, et al., 2023; Xie, Zhao, et al., 2023), thereby forming an antioxidant network composed of phenolic compounds, nitrogen compounds, vitamins, and other free radical scavenging molecules to reduce ROS levels (Agati et al., 2012; Cai et al., 2003; Chiang et al., 2013; Wong et al., 2006). These natural antioxidants are isolated from plants and characterized, and are found to play an excellent role in anti‐cancer; for example, natural antioxidants isolated from plants such as *Tetradesmus acuminatus* microalgae and artichoke showed an inhibitory effect on breast cancer (Mileo et al., 2020; Mujeeb et al., 2020). Studies have shown that garlic has physiological activities such as anti‐inflammatory (Gruhlke et al., 2016; Schafer & Kaschula, 2014), anti‐cancer (Kim et al., 2018; Tsubura et al., 2011), and anti‐oxidation (Shang et al., 2019; Wilson & Demmig‐Adams, 2007). However, garlic peels are often discarded as agricultural waste for garlic processing, causing a waste of resources. Previously, Ichikawa et al. demonstrated that the phenols (all phenylpropanoids) in garlic peel extract are the main components of their antioxidant activity, and characterized six phenols (Ichikawa et al., 2003). However, the relationship between garlic peel extract as an antioxidant and the occurrence and development of cancer remains to be elucidated.

The objective of this study was to assess the antioxidant properties of garlic peel extract, examine its inhibitory effects on 4T1 tumors in mice with triple‐negative breast cancer, and elucidate the potential mechanisms underlying its tumor‐inhibiting properties. This research aims to enhance our comprehension of the biological functions of garlic peel extract and its potential therapeutic applications in cancer treatment.

## 2 MATERIALS AND METHODS

### 2.1 Sample and sample preparation

Garlic (*Allium sativum L*.) was cultivated in Yantai, Shandong, China, during the 2019 growing season and harvested in August. The extraction of garlic peel was conducted using a previously reported method (Ichikawa et al., 2003).

### 2.2 Ultra‐high performance liquid chromatography‐mass spectrometry/mass spectrometry (UPLC‐MS/MS)

#### 2.2.1 Sample preparation and extraction

The samples underwent freeze‐drying using a vacuum freeze‐dryer (Scientz‐100F) and were subsequently pulverized in a mixer mill (MM 400, Retsch) with a zirconia bead for 1.5 min at 30 Hz. Following this, 100 mg of lyophilized powder was dissolved in 1.2 mL of a 70% methanol solution, vortexed for 30 s every 30 min for a total of 6 cycles, and then stored in a refrigerator at 4°C overnight. The resulting mixture was then centrifuged at 12000 rpm for 10 min, and the extracts were filtered through a 0.22 μm pore size filter (SCAA‐104) prior to UPLC‐MS/MS analysis.

#### 2.2.2 Identification and quantification of polyphenol compounds

Sample extracts were identified through UPLC‐ESI‐MS/MS analysis (UPLC, SHIMADZU Nexera X2; MS, Applied Biosystems 4500 Q TRAP), which was conducted using an Agilent SB‐C18 column (1.8 μm, 2.1 *×* 100 mm) with a mobile phase consisting of pure water with 0.1% formic acid (solvent A) and acetonitrile with 0.1% formic acid (solvent B). The column oven was maintained at 40°C, with a 4 μL sample injection and a flow rate of 0.35 mL/min. The effluent was subjected to analysis utilizing an ESI‐triple quadrupole‐linear ion trap (QTRAP)‐MS/MS system, operating in both positive and negative ion detection modes.

### 2.3 Determination of peroxyl radical scavenging capacity (PSC)

The PSC assay was performed in accordance with the methodology described in a prior publication (Adom & Liu, 2005). Briefly, PSC was measured using an antioxidant assay involving a sample extract of Vitamin C (Vc) mixed with dichlorofluorescein (DCFH) and 2,2′‐Azobis (2‐amidinopropane) dihydrochloride (ABAP) at 37°C. Fluorescence was measured at 538 nm emission every 2 min for 40 min using a Fluoroskan Ascent fluorescent spectrophotometer (Thermo Labsystems, MA, USA). PSC was presented as mg of Vc equiv. (VCE)/100 g DW.

### 2.4 Determination of cellular antioxidant activity (CAA)

The CAA assay was performed in accordance with the methodology described in a prior publication (Wolfe & Liu, 2007). Briefly, after 24 h of incubation, the growth medium was removed, and HepG2 cells were washed with PBS. Then, antioxidant treatment medium with DCFH‐DA and varying concentrations of phenolic extracts was added to triplicate wells. After 1 h, the treatment medium was removed. Cells were either washed once with PBS or not washed at all. A fresh solution of 600 μM ABAP in an oxidant treatment solution was then added to the cells. Fluorescence emission at 538 nm was measured every 5 min for 1 h using an excitation at 485 nm. The CAA of the phenolic extract was expressed as μmol quercetin equiv. (QE)/100 g DW.

### 2.5 4T1 cell culture and tumor‐bearing mouse establishment

The 4T1 breast cancer cell line was obtained from the Cell Bank of the Chinese Academy of Sciences and cultured at 37°C with 5% CO_2_ (Li, Zheng, et al., 2021; Lu et al., 2019). Female BALB/c mice aged 6–8 weeks were purchased from Changsheng Animal Resources Center and kept at Shenyang Agricultural University. All animal experiments were approved by the Committee on the Ethics of Animals of Shenyang Agricultural University (Permit Number: SYXK 2021‐0010). To assess the anticancer efficacy against orthotopic TNBC in vivo, 2 × 10^5^ cells were implanted into the fourth mammary fat pad of BALB/c mice. Seven‐day post‐transplantation, mice were randomly divided into four groups of five animals each and treated with garlic peel extract, including a health, 4T1 (saline i.p.), 4T1 plus garlic peel extract (20 mg/kg bw/d i.p.), and 4T1 plus garlic peel extract (100 mg/kg bw/d i.p.). The dimensions of the tumor, including width (*W*) and length (*L*), were assessed using calipers at 3‐day intervals to track the overall tumor volume (mm^3^), which was determined using the formula *V* = 1/2 × *L* × *W*2. All experimental mice were euthanized at the 14‐day mark post‐transplantation to minimize any unnecessary suffering or distress. Serum levels of alanine aminotransferase (ALT) and aspartate aminotransferase (AST) were quantified using commercial assay kits (Sigma) following the manufacturer's guidelines.

### 2.6 Histology and immunohistochemistry (IHC)

Tumors and livers were weighed and fixed in a 4% paraformaldehyde solution for subsequent histological analysis. Following dissection, the tumors and livers underwent histological processing, which commenced with an overnight fixation in a 4% paraformaldehyde solution (Solarbio, cat. No P1110) at 4°C. Subsequently, all samples were subjected to blind review by pathologists for diagnosis. The immunohistochemical method is consistent with that described in the previous article (Allred et al., 1998). The primary antibody was diluted to the appropriate concentration as follows: COX2/Cyclooxygenase 2 (ab15191, Abcam, Cambridge, UK; 1:2000), platelet endothelial cell adhesion molecule‐1 (CD31/ PECAM‐1, ab182981; Abcam; 1:2000), vascular endothelial growth factor A (VEGFA, BA0407; Boshide; 1:200), matrix metalloproteinase 2 (MMP2, A6247; ABclonal; 1:100), and matrix metalloproteinase 9 (MMP9, A0289; ABclonal; 1:100). Five different images per slide from 15 random slides were randomly analyzed for each group for immunohistochemical quantification. The level of positive expression was assessed utilizing Image‐Pro Plus 6.0 software (Media Cybernetics, Silver Spring, MD, USA) and was reported as the mean ± SE (standard error) (Mi et al., 2018).

### 2.7 Statistical analysis

The Student's t‐test was employed to assess the significance of the disparity between the groups, with a p‐value of less than 0.05 being deemed statistically significant. All statistical analyses were conducted utilizing the R statistical software, version 3.50.

## 3 RESULTS

### 3.1 Characterization of garlic peel extract

**FIGURE 1:**
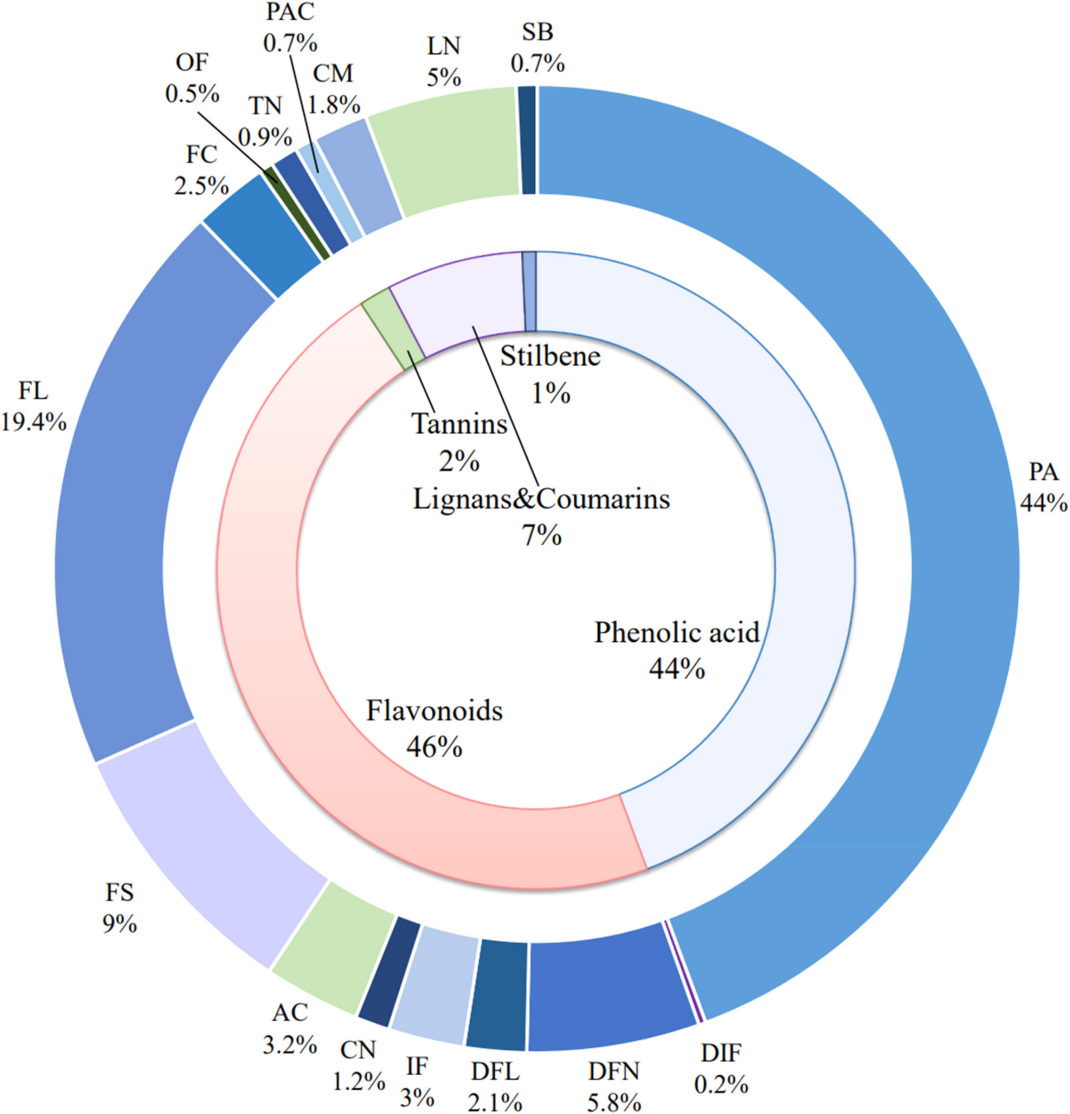
The number and type of polyphenols in garlic peel. Phenolic acids (PA), dihydroisoflavones (DIF), flavanones (DFN), flavanonols (DFL), isoflavones (IF), chalcones (CN), anthocyanidins (AC), flavones (FS), flavonols (FL), flavonoid carbonoside (FC), other flavonoids (OF), tannins (TN), proanthocyanidins (PAC), coumarins (CM), lignans (LN), and stilbene (SB).

In order to clarify the antioxidant potential of garlic peel extract, extensively targeted metabolomics was applied. The metabolites of garlic peel extract were analyzed based on the UPLC‐MS/MS. As shown in Figure S1, 187 and 246 peaks were identified in positive and negative ionization modes, respectively. A total of 433 polyphenol compounds were finally identified, including 192 phenolic acids, 84 flavonols, 39 flavonoids, 25 flavanones, 14 anthocyanidins, 11 isoflavones, 11 flavonoid carbonosides, 9 flavanonols, 5 chalcones, 2 other flavonoids, 1 dihygroisoflavone, 22 lignans, 8 coumarins, 4 tannins, 3 proanthocyanidins, and 3 stilbenes (Figure 1). Flavonoids (46%) and phenolic acid (44%) are the main polyphenols present in the extracts of garlic peel extract; flavonols are the main contributors of flavonoids, accounting for 19.4%.

### 3.2 The antioxidant activity of garlic peel extract measured by PSC and CAA assays

As shown in Table 1, the free‐, bound‐, and total PSC of garlic peel extract were 5702.80 ± 102.63, 895.27 ± 17.00, and 6598.07 ± 119.63 μmol VCE/100 g FW, respectively. Among them, free PSC was the major contributor to total PSC in the garlic peel extract (86.43%), while bound PSC was about 13.57% of total PSC. The CAAs of free and bound phenolics are shown in Table 1. In the absence of a PBS wash, the CAA assay assesses the antioxidant activities of compounds both bound to the cell membrane and those entering the cells. Conversely, in the presence of PBS wash, the CAA assay only measures the antioxidant activities of compounds entering the cells, as the compounds bound to the cell membrane are removed by PBS (Wolfe & Liu, 2007). In the free CAA protocol, the cellular intake rate (calculated as the difference between the PBS value and the no PBS value) was found to be 15.41%, whereas the cellular uptake rate in the bound CAA protocol was 13.59%.

**TABLE 1:** Antioxidant activity of garlic peel extract measured by peroxyl radical scavenging capacity (PSC) and cellular antioxidant activity (CAA) assay.

Antioxidant activity	Free	Bound	Total
PSC (μmol VCE/100 g FW)	5702.80 ± 102.63^a^	895.27 ± 17.00^b^	6598.07 ± 119.63^c^
PBS wash CAA (μmol QE/100 g FW)	73.54 ± 4.03^a^	1.68 ± 0.15^b^	75.22 ± 3.14
No PBS wash CAA (μmol QE/100 g FW)	477.30 ± 34.25^a^	12.36 ± 1.59^b^	490.66 ± 5.50^c^

^a^ Corresponds to the comparison of free and bound group.

^b^ Corresponds to the comparison of bound and total group.

^c^ Corresponds to the comparison of free and total group. Differences were considered significant at *p* < .05. Results are expressed as mean ± SD (*n* = 3).

### 3.3 Inhibitory effect of garlic peel extract on 4T1 tumor growth in situ

**FIGURE 2:**
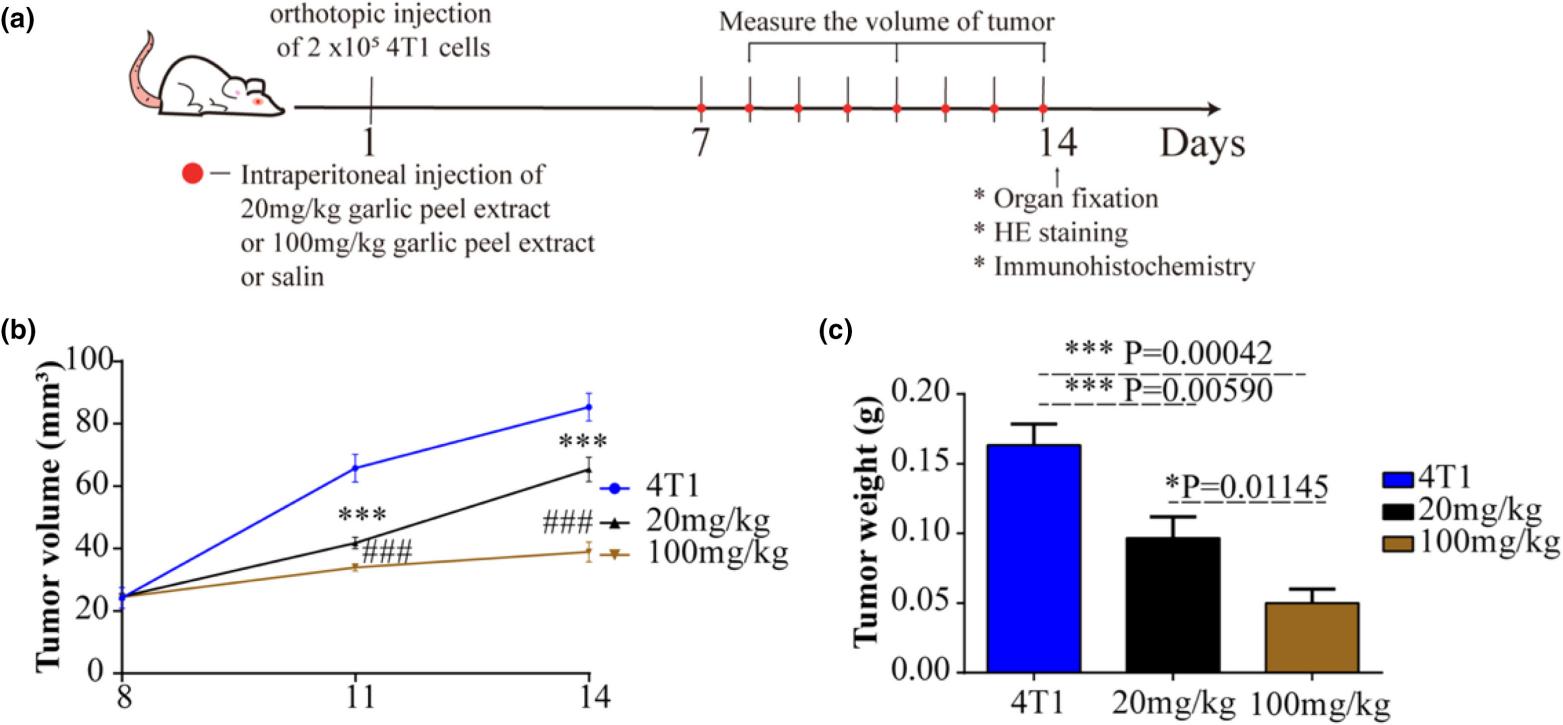
(a) Experimental design. (b) The tumor volume. (c) The tumor weight (wet) on the 14th day. The mean difference was compared by *t*‐test (*p* < .05). * indicates that the *p* value is less than .05. ** indicates that the *p* value is less than .01. 4T1 = 4T1 tumor‐bearing mice by intraperitoneal injection of the same volume of saline; 20 mg/kg = 4T1 tumor‐bearing mice by intraperitoneal injection of 20 mg/kg garlic peel extract. 100 mg/kg = 4T1 tumor‐bearing mice by intraperitoneal injection of 100 mg/kg garlic peel extract. These results have shown an average of ± SEM.

The experimental design is shown in Figure 2a. Firstly, 2 × 10^5^ cells were in situ injected into the fourth pair of mammary glands of mice. When the tumor grew to the 7th day, the 4T1 tumor‐bearing mice were injected intraperitoneally with low‐concentration peel extract (20 mg/kg), high‐concentration (100 mg/kg) garlic peel extract, or normal saline for 7 days, and the tumor volume was measured. On the 14th day, the mice were killed, and their organs and peripheral blood were collected. It can be seen from Figure 2b that both high and low concentrations of garlic peel extract can inhibit the growth of the 4T1 tumor, and the high concentration group has a more significant effect on tumor growth. The results of tumor weight are consistent with those of tumor volume (Figure 2c), indicating that garlic peel extract has an inhibitory effect on 4T1 in situ tumors and that the inhibitory effect is dose‐dependent.

### 3.4 Alterations of liver pathological and biochemical indexes in 4T1 tumor‐bearing mice

**FIGURE 3:**
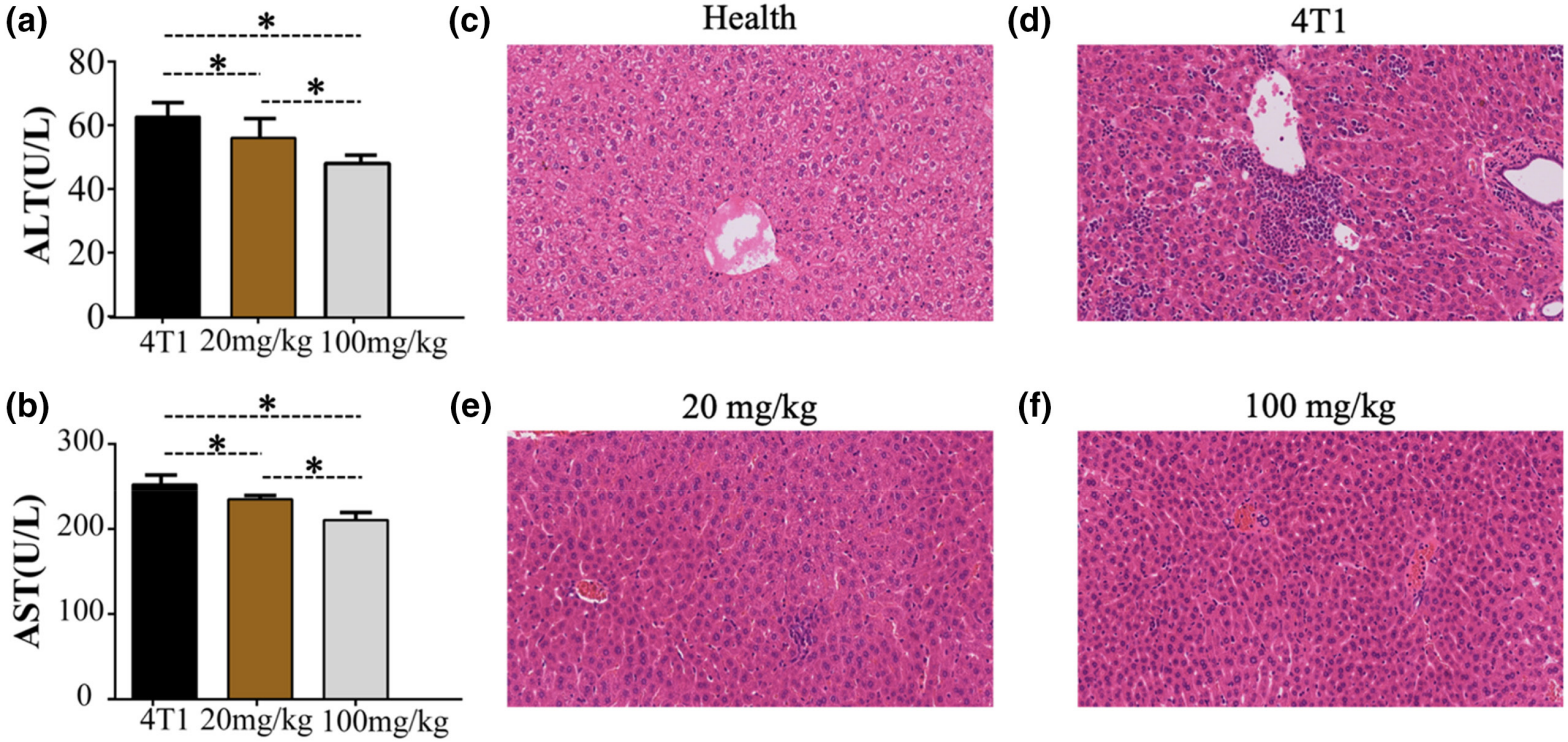
The content of ALT in serum (a) the content of AST in serum (b) representative HE photos of the liver of mice intraperitoneally injected with the same volume of saline, 20 mg/kg garlic peel extract, and 100 mg/kg garlic peel extract (c) the representative photos were taken at ×200 magnification. 4T1 = 4T1 tumor‐bearing mice by intraperitoneal injection of the same volume of saline; 20 mg/kg = 4T1 tumor‐bearing mice by intraperitoneal injection of 20 mg/kg garlic peel extract. 100 mg/kg = 4T1 tumor‐bearing mice by intraperitoneal injection of 100 mg/kg garlic peel extract. Health = Healthy mice without any treatment. Scale = 100 μm. These results have shown an average of ± SEM.

In order to investigate whether garlic peel extract treatment could induce liver damage in mice, the biochemical markers of liver injury in mice were determined, and the pathological changes of liver tissue in mice were detected by typical HE staining. Biochemical analysis showed that, compared with the 4T1 group, the ALT (Figure 3a) and AST (Figure 3b) in the high and low concentration groups were decreased, and the values in the high concentration group were lower, indicating that garlic peel extract can protect the liver from liver injury caused by the 4T1 tumor. The protective effect of garlic peel extract on the liver of 4T1 tumor‐bearing mice was further explored by histopathological results. As shown in Figure 3c, the liver cords of the healthy mice group were neatly arranged, there was no necrosis in the hepatic lobules, and there was no red blood cell aggregation in the portal vein of the liver. In contrast to the control group, the hepatic cord arrangement in the 4T1 group (Figure 3d) exhibited increased disorganization, accompanied by a higher concentration of red blood cells within the hepatic vein. The hepatic cord was arranged neatly in the low concentration group (20 mg/kg) compared with the 4T1 group, but the hepatic vein congestion was not alleviated. Compared with the 4T1 group, the high concentration group (100 mg/kg) arranged the hepatic cord more neatly and reduced the hepatic vein congestion and scattered inflammatory cell infiltration.

### 3.5 Inhibitory effect of garlic peel extract on COX‐2 expression in 4T1 tumor tissue

**FIGURE 4:**
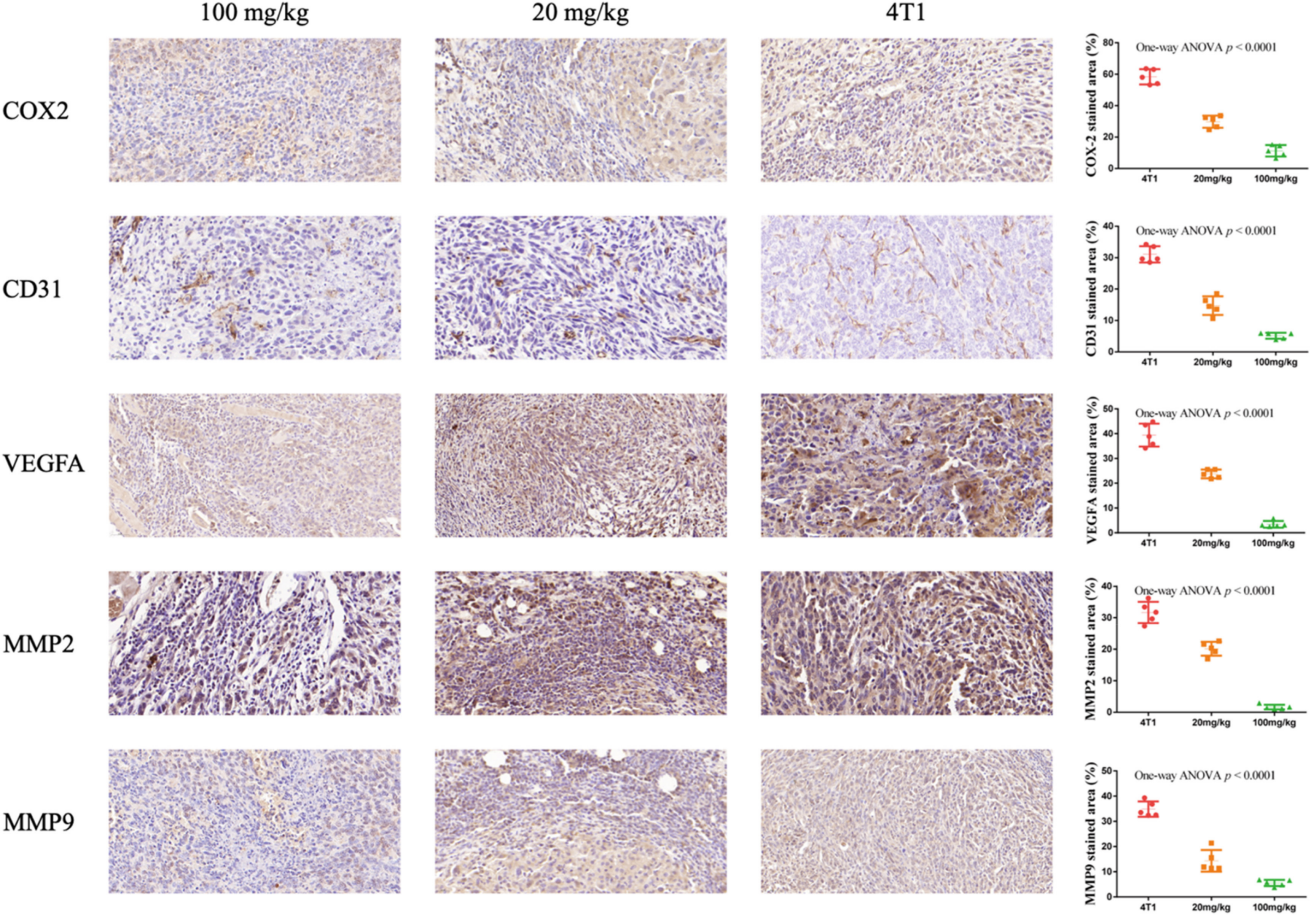
Representative immunohistochemistry of COX‐2, CD31, VEGFA, MMP2, and MMP9. The representative tumor sections taken at ×200 magnifications are shown. The column chart shows the stained area; semiquantitative analysis of COX‐2, CD31, VEGFA, MMP2, and MMP9 expression in tumors to IHC. 4T1 = 4T1 tumor‐bearing mice by intraperitoneal injection of the same volume of saline; 20 mg/kg = 4T1 tumor‐bearing mice by intraperitoneal injection of 20 mg/kg garlic peel extract. 100 mg/kg = 4T1 tumor‐bearing mice by intraperitoneal injection of 100 mg/kg garlic peel extract. Scale = 100 μm.

Figure 4 illustrates that the expression of COX‐2 was reduced in both treatment groups. In addition, the high‐concentration group (100 mg/kg) more significantly inhabited the COX‐2 expression compared with the low‐concentration group (20 mg/kg). This suggests that garlic peel extract has the potential to hinder tumor growth by suppressing COX‐2 expression in tumor tissues, thereby highlighting the imbalance between reactive oxygen generation and antioxidant defense in 4T1 tumors. Studies have shown that COX‐2 expression is positively correlated with CD31 and VEGFA expression in breast cancer (Timoshenko et al., 2006). It can be seen that the immunohistochemical results of CD31 and VEGFA are consistent with the expression of COX‐2. The group administered with a high concentration (100 mg/kg) of garlic peel extract demonstrated a significant inhibition of CD31 and VEGFA expression in 4T1 tumor tissues. Conversely, the group receiving a low concentration (20 mg/kg) also exhibited inhibition of CD31 and VEGFA expression, albeit to a lesser extent compared to the high‐concentration group. Furthermore, the expression of MMP2 and MMP9 in 4T1 tumor tissue was investigated, with immunohistochemical findings indicating a correlation with COX‐2 expression. These results suggest that garlic peel extract has a dose‐dependent inhibitory effect on MMP2 and MMP9 expression in 4T1 tumors.

## 4 DISCUSSION

Studies have shown that some antioxidant properties of plant components are closely related to human health (Losada‐Echeberría et al., 2017), so it is particularly important to choose appropriate methods to evaluate the antioxidant activity of garlic peels. First, the PCS assay was used to assess the peroxyl radical scavenging capacity of garlic peel extract. Compared with other traditional methods such as DPPH, ABTS, and FRAP, the PCS assay is a more simple, rapid, and sensitive assay and considers the naturally existing reactive oxygen radicals and physiological conditions (pH and temperature) in the body (Adom & Liu, 2005; Xiang et al., 2019). Previously, Ichikawa et al. explored the DPPH radical scavenging activity of garlic peel extract and found that strong activity eliminated ∼90% of the DPPH radicals at a concentration of 0.1% (Ichikawa et al., 2003). In this study, the peroxyl radical scavenging capacity of garlic peel extract was re‐evaluated using the PCS assay, including both free and bound components, which would provide new insights into the antioxidant activity of garlic peel. The free PSC value of garlic peel extract (5702.80 ± 102.63 μmol VCE/100 g FW) was significantly higher than that of grape (2018.9 μmol VCE/100 g FW), cranberry (1019.9 μmol VCE/100 g FW), apple (309.2 μmol VCE/100 g FW), and kiwifruit (17.48–55.58 μmol VCE/100 g FW) (Adom & Liu, 2005; Saeed et al., 2019). Our results showed that free fractions of garlic peel extract had the ability to scavenge free radicals efficiently, and thereby could be used as antioxidants for disease prevention and control. The bound PSC value of garlic peel extract was 895.27 ± 17.00 μmol VCE/100 g FW, which was significantly lower than that of the free PSC value, indicating that the total phenolic contents in free fractions were significantly higher than in the bound fractions in garlic peel extract. The cellular antioxidant capacity of garlic peel extract was assessed using the CAA assay, which is considered a more biologically relevant method compared to traditional chemical antioxidant assays. This assay is valuable for investigating the bioavailability of food antioxidants due to its consideration of cellular uptake and metabolism processes (Kellett et al., 2018). Moreover, the antioxidants employed in the cellular antioxidant activity (CAA) assay demonstrate the ability to be readily absorbed into cells, where they can either interact with reactive oxygen species (ROS) intracellularly or disrupt peroxyl radical chain reactions at the cell membrane (Günther et al., 2007). The resulting CAA values show promise for correlating with in vivo antioxidant activity. The CAA assessment, conducted following a phosphate‐buffered saline (PBS) wash protocol, highlights the potential of phytochemicals to penetrate cell membranes. Notably, the reduced fluorescence observed with garlic peel extract suggests a significant antioxidant capacity compared to the control group. The findings of this study demonstrated that garlic peel exhibits potent antioxidant properties, suggesting its potential utility as a viable source for the development of functional materials.

Previously, a study showed that six polyphenols in garlic peel extracts are the main components of their antioxidant activity (Ichikawa et al., 2003). Based on this, this study further detected 16 types of polyphenols in garlic peels via UPLC‐MS/MS, including phenolic acid (44%), flavonols (19.4%), flavonoids (9%), flavanones (5.8%), etc. Metabolomics results showed that the antioxidative ability of garlic peel is attributed to flavonoids and polyphenols. Garlic peel in this study has a high content of quercetin, catechin, ferulic acid, and p‐coumaric acid, which is consistent with previous studies (Ichikawa et al., 2003; Ullah et al., 2020). These natural bioactive compounds are the most widespread plant polyphenols due to their antioxidant ability to scavenge free superoxide radicals and reduce the risk of cancer (Ghasemzadeh & Ghasemzadeh, 2011). Ji et al.'s study illustrated that the hippophae extract has high flavonoids and phenolic acid content with the same antiproliferative and proapoptotic effect as garlic peel on breast cancer (Ji et al., 2020). Moreover, metabolomics results in our study found flavanols are the main flavonoids in garlic peels. A meta‐analysis of breast cancer suggested that flavanols intake is significantly associated with the reduction of breast cancer risk compared with other flavonoid subclasses or total flavonoids (Hui et al., 2013). Therefore, investigating the flavanols of garlic peel could be a promising study orientation with potential beneficial applications in the treatment of breast cancer.

Additionally, our results showed that the garlic peel extracts of both high (100 mg/kg) and low (20 mg/kg) concentration groups can inhibit the growth of the 4T1 tumor in mice, and the inhibitory effect of the high concentration group is more significant. Polyphenols in other plant sources also have the same effect on inhibiting breast cancer growth as garlic peel extract. Studies have shown that ephedra extract has an inhibitory effect on the 4T1 tumor and can enhance the anti‐proliferative and pro‐apoptotic capabilities of cisplatin (Sioud et al., 2020). Günther et al. demonstrated that polyphenols can reduce ROS levels in 4T1 tumors, inhibit MMP9 expression, and inhibit tumor metastasis (Günther et al., 2007). Similarly, studies reported that myricetin and epigallocatechin‐3‐gallate can repress the migration, metastasis, invasion, and adhesion of TNBC cells by reducing the MMP‐2/9 protein expression (Adinew et al., 2021).These plant‐derived polyphenols play an anti‐cancer role as antioxidants, which provides a direction for the development of functional plant‐based foods and also provides a theoretical basis for the screening of anti‐cancer drugs and research on potential targets.

A previous study has shown that COX‐2 is overexpressed in 40% of invasive breast cancers and is closely related to proliferation, histological grading, metastasis, and survival (Howe, 2007). COX‐2 overexpression contributes to tumor progression and metastasis through multiple mechanisms (Singh et al., 2007). During this complex regulation, COX‐2 activity modulates other proteins, such as MMP2, MMP9, and VEGFA, thus promoting the molecular mechanism of cell invasion (Sivula et al., 2005; von Rahden et al., 2005). Enhanced prostaglandin production is the direct result of COX‐2 activity, and it can activate downstream signaling cascades such as the MAPK/ERK pathway, thus increasing MMP expression (Liou & Storz, 2010; Sivula et al., 2005). VEGF expression is also upregulated by COX‐2 through the prostaglandin signaling pathway, and VEGF overexpression can provide tumors with nutrients and oxygen (Davies et al., 2003). Our study showed that both high and low concentrations of garlic peel extract can inhibit the expression of COX‐2 in the 4T1 tumor, and the inhibitory effect of the high concentration group is more significant. In this study, it has also been found that the expression of CD31 and VEGFA in 4T1 tumor tissues was inhibited by garlic peel extract and was positively correlated with the expression of COX‐2, which is consistent with the findings of previous studies (Timoshenko et al., 2006). Other than this, the expression of MMP2 and MMP9 in 4T1 tumor tissues was inhibited by garlic peel extracts and was positively correlated with the expression of COX‐2. These results suggested that garlic peel extract can inhibit the prostaglandin signaling pathway by reducing the expression of COX‐2, VEGFA, and MMP, thereby suppressing the metastasis and angiogenesis of breast cancer progression. However, the mechanisms of COX‐2‐induced cancer cell metastasis are multiplex and equivocal; it is not clearly defined whether garlic peel directly or indirectly inhibits COX‐2 expression; this needs to be explored in future studies.

## 5 CONCLUSIONS

In this study, 16 types of polyphenol compounds have been identified; flavonoids and polyphenols are the main antioxidants from garlic peels. In addition, this study assessed the antioxidant activity of garlic peel extract through the employment of PSC and CAA methods, examined the suppressive impact of garlic peel extract on 4T1 orthotopic tumor growth, and demonstrated that both high and low concentration groups exhibited inhibitory effects. Furthermore, a plausible mechanism for the antioxidant properties of garlic peel extract in inhibiting 4T1 tumor growth and angiogenesis was elucidated, specifically through the inhibition of COX‐2 expression. Our findings offer a theoretical foundation for the utilization of garlic by‐products in the food industry and the advancement of functional garlic peel products. Moreover, the investigation of natural antioxidants may provide a novel idea for ameliorating breast cancer therapy.

## AUTHOR CONTRIBUTIONS

**Yushi Dong:** Formal analysis (equal); investigation (equal); methodology (equal); validation (equal); visualization (equal); writing – original draft (equal). **Jiyue Zhang:** Data curation (equal); investigation (equal); resources (equal); visualization (equal). **Aijun Xie:** Formal analysis (equal); writing – review and editing (equal). **Xiqing Yue:** Investigation (equal); methodology (equal); writing – review and editing (equal). **Mohan Li:** Conceptualization (equal); funding acquisition (equal); investigation (equal); project administration (equal); supervision (equal); visualization (equal); writing – original draft (equal); writing – review and editing (equal). **Qian Zhou:** Methodology (equal); resources (equal); supervision (equal); writing – review and editing (equal).

## CONFLICT OF INTEREST STATEMENT

None.

## Supporting information


Figure S1.

## Data Availability

The data that support the findings of this study are available on request from the corresponding author.
