# Supplementary material for: Garlic peel extract as an antioxidant inhibits triple‐negative breast tumor growth and angiogenesis by inhibiting cyclooxygenase‐2 expression
Source: Food Sci Nutr. 2024 Jul 9;12(9):6886–95. doi: 10.1002/fsn3.4320 (PMC11561839; doi:10.1002/fsn3.4320)
Supplement: Supplementary file 1 — Figure S1. [file FSN3-12-6886-s001.docx]

**Supplementary Figure**

**Supplementary Fig. 1.** The total ions current of garlic peel extract. Negative ion mode (A). Positive ion mode (B).


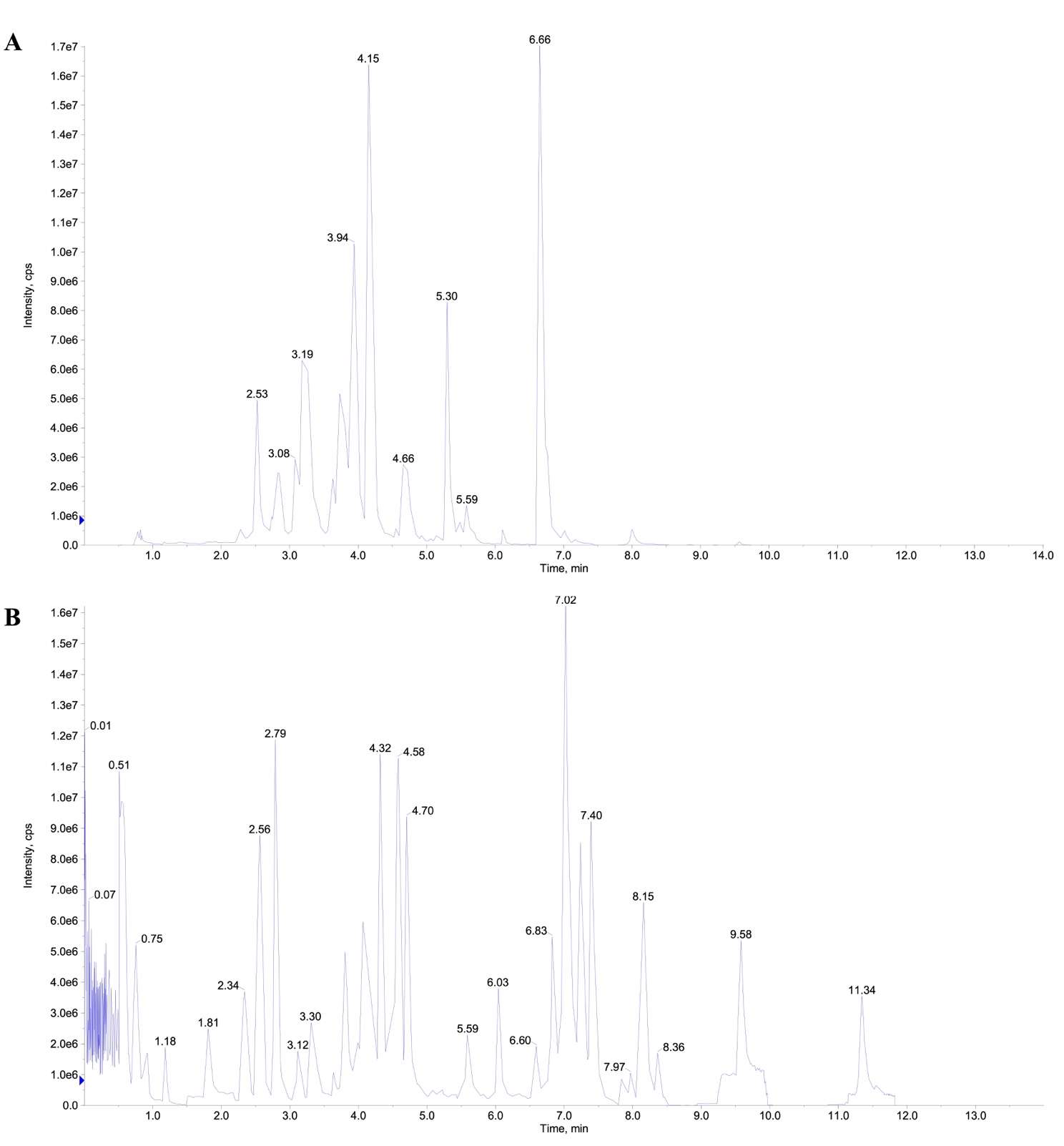


**Supplementary Fig. 1.** The total ions current of garlic peel extract. Negative ion mode (A). Positive ion mode (B).
